# Rapid Rebound of the Treg Compartment in DEREG Mice Limits the Impact of Treg Depletion on Mycobacterial Burden, but Prevents Autoimmunity

**DOI:** 10.1371/journal.pone.0102804

**Published:** 2014-07-22

**Authors:** Luciana Berod, Philipp Stüve, Filipa Varela, Jochen Behrends, Maxine Swallow, Friederike Kruse, Freyja Krull, Peyman Ghorbani, Christian T. Mayer, Christoph Hölscher, Tim Sparwasser

**Affiliations:** 1 Institute of Infection Immunology, TWINCORE, Centre for Experimental and Clinical Infection Research, a Joint Venture between the Medical School Hannover (MHH) and the Helmholtz Centre for Infection Research (HZI), Hannover, Germany; 2 Priority Research Area "Infection", Division "Infection Immunology", Research Center Borstel, Borstel, Germany; 3 Core Facility "Fluorescence Cytometry", Research Center Borstel, Borstel, Germany; 4 Cluster of Excellence "Inflammation at Interfaces", Christian-Albrechts-University, Kiel, Germany; Institut Pasteur, France

## Abstract

The development of an effective vaccine against tuberculosis (Tb) represents one of the major medical challenges of this century. *Mycobacterium bovis* Bacille Calmette-Guerin (BCG), the only vaccine available at present, is mostly effective at preventing disseminated Tb in children, but shows variable protection against pulmonary Tb, the most common form in adults. The reasons for this poor efficacy are not completely understood, but there is evidence that T regulatory cells (Tregs) might be involved. Similarly, Tregs have been associated with the immunosuppression observed in patients infected with Tb and are therefore believed to play a role in pathogen persistence. Thus, Treg depletion has been postulated as a novel strategy to potentiate *M. bovis* BCG vaccination on one side, while on the other, employed as a therapeutic approach during chronic Tb infection. Yet since Tregs are critically involved in controlling autoimmune inflammation, elimination of Tregs may therefore also incur the danger of an excessive inflammatory immune response. Thus, understanding the dynamics and function of Tregs during mycobacterial infection is crucial to evaluate the potential of Treg depletion as a medical option. To address this, we depleted Tregs after infection with *M. bovis* BCG or *Mycobacterium tuberculosis* (*Mtb*) using DEREG mice, which express the diphtheria toxin (DT) receptor under the control of the FoxP3 locus, thereby allowing the selective depletion of FoxP3^+^ Tregs. Our results show that after depletion, the Treg niche is rapidly refilled by a population of DT-insensitive Tregs (diTregs) and bacterial load remains unchanged. On the contrary, impaired rebound of Tregs in DEREG × FoxP3^GFP^ mice improves pathogen burden, but is accompanied by detrimental autoimmune inflammation. Therefore, our study provides the proof-of-principle that, although a high degree of Treg depletion may contribute to the control of mycobacterial infection, it carries the risk of autoimmunity.

## Introduction

Intervention strategies to fully eradicate tuberculosis (Tb) comprise two main aspects: the prevention of new cases with effective vaccines and the therapeutic treatment of already infected patients. T regulatory cells (Tregs) represent a population of CD4^+^ T cells whose main function is to control autoimmunity and prevent excessive inflammatory responses that can be detrimental to the host [1], [2]. During infection, Tregs can control immune-mediated pathology by negatively regulating effector immune mechanisms against the invading pathogen [3], [4]. However, this negative feedback can also contribute to inefficient microbial clearance and the establishment of chronic infections [4], [5]. Indeed, depletion of Tregs during chronic viral infection has been shown to allow reactivation of exhausted CD8^+^ T cells and clearance of infection [6]. Moreover, Treg expansion is considered a mechanism of immune evasion that can be directly exploited by certain pathogens. On the one hand, Tregs appear to be capable of sensing pathogen-associated molecular patterns via Toll-like receptor 2 (TLR2), and probably other not-yet identified pattern recognition receptors [7]. On the other hand, Tregs can also be indirectly modulated by environmental cues from immune and non-immune cells [8]. Infection with *Mycobacterium tuberculosis* (*Mtb*) has been shown to induce the expansion of Tregs both in mice [9] and humans [10]–[12]. Yet, it is not clear whether this is exclusively a direct effect of mycobacteria on Tregs or also a consequence of the highly inflammatory environment. After infection with *Mtb*, Tregs are not only present in lymphoid organs, but are also recruited to lung granulomas in a TLR2-dependent manner [9], [13]. Likewise, Treg expansion has been also observed upon *M. bovis* Bacille Calmette-Guerin (BCG) vaccination and there is evidence that Tregs may correlate with the poor efficiency of this vaccine strain in conferring protection from pulmonary Tb [14], [15]. Particularly, BCG efficacy is lower in developing countries, where people are continuously exposed to low levels of environmental mycobacteria or helminth infections, both associated with high number of circulating Tregs [16], [17]. As a consequence, it has been proposed that strategies allowing for the manipulation of Tregs may represent a powerful tool for preventing or treating Tb infection.

Tregs are characterised by the specific expression of the transcription factor FoxP3, which is essential for their immunosuppressive phenotype [18]–[20]. Though FoxP3 is the most widely used Treg marker, studies that specifically target this factor to deplete Tregs were hindered due to its intracellular expression. Initial reports have mainly relied on anti-CD25 antibodies to deplete Tregs, a rather unspecific strategy since it also eliminates effector immune cells and some Tregs can downregulate CD25 expression. Using this approach, elimination of Tregs before *M. bovis* BCG or *Mtb* infection resulted in an increased IFN-γ response, but only marginal or transient effects on bacterial load [21], [22]. At the same time, adoptive transfer of a Treg/T effector cell mixture lead to an increased bacterial burden in *Mtb* infected mice compared to transfer of T effector cells alone [23] and depletion of Tregs in bone marrow chimeras was beneficial for controlling mycobacteria in the lungs but not in the spleen [9]. Yet, in the latter model, permanent depletion of Tregs caused strong immune activation that was independent of the infection and resembled autoimmune disease. In addition, it is not clear whether Treg depletion during an established infection can be used as a therapeutic option to treat *Mtb* infection. Depletion of Tregs during the priming phase of an immune response can affect the pool of memory T cells [24], whereas depletion during the later course of infection expands short lived effector T cells [25]. Therefore, the timing of Treg depletion may be critical for the long-term protection conferred by this strategy.

To evaluate the potential of Treg depletion as a therapeutic tool against mycobacterial infection, we used DEREG BAC transgenic FoxP3 reporter mice, which express a fusion molecule of the diphtheria toxin receptor (DTR) and eGFP (enhanced green fluorescent protein) under the control of the FoxP3 locus, allowing the specific *in vivo* depletion of FoxP3^+^ Treg cells during the course of infection [26], [27]. Since adult DEREG mice do not develop autoimmunity after Treg depletion as it was observed in other mouse strains [28]–[30], this model is suitable to study the role of Tregs during infection without the side effects of autoimmune activation, as it was demonstrated in several models of chronic infection [6], [25], [31]–[33].

While previous reports addressing the role of Tregs in Tb have used *M. bovis* BCG or *Mtb*, we here examined the effect of Treg depletion in DEREG mice in both models [9], [15], [21]. Our results show that depletion of eGFP^+^FoxP3^+^ Tregs using DEREG mice after *M. bovis* BCG or *Mtb* infection leads to a rapid rebound of the Treg pool, mostly at the expense of DT-insensitive eGFP^−^FoxP3^+^ Treg cells (diTregs). On the contrary, depletion of Tregs in *M. bovis* BCG infected DEREG × FoxP3^GFP^ mice, a model with impaired Treg homeostasis after depletion, results in slightly decreased bacterial burden. However, this effect was associated with massive autoimmune activation.

## Results

### Limited expansion of Tregs upon *M. bovis* BCG infection

**Figure 1:**
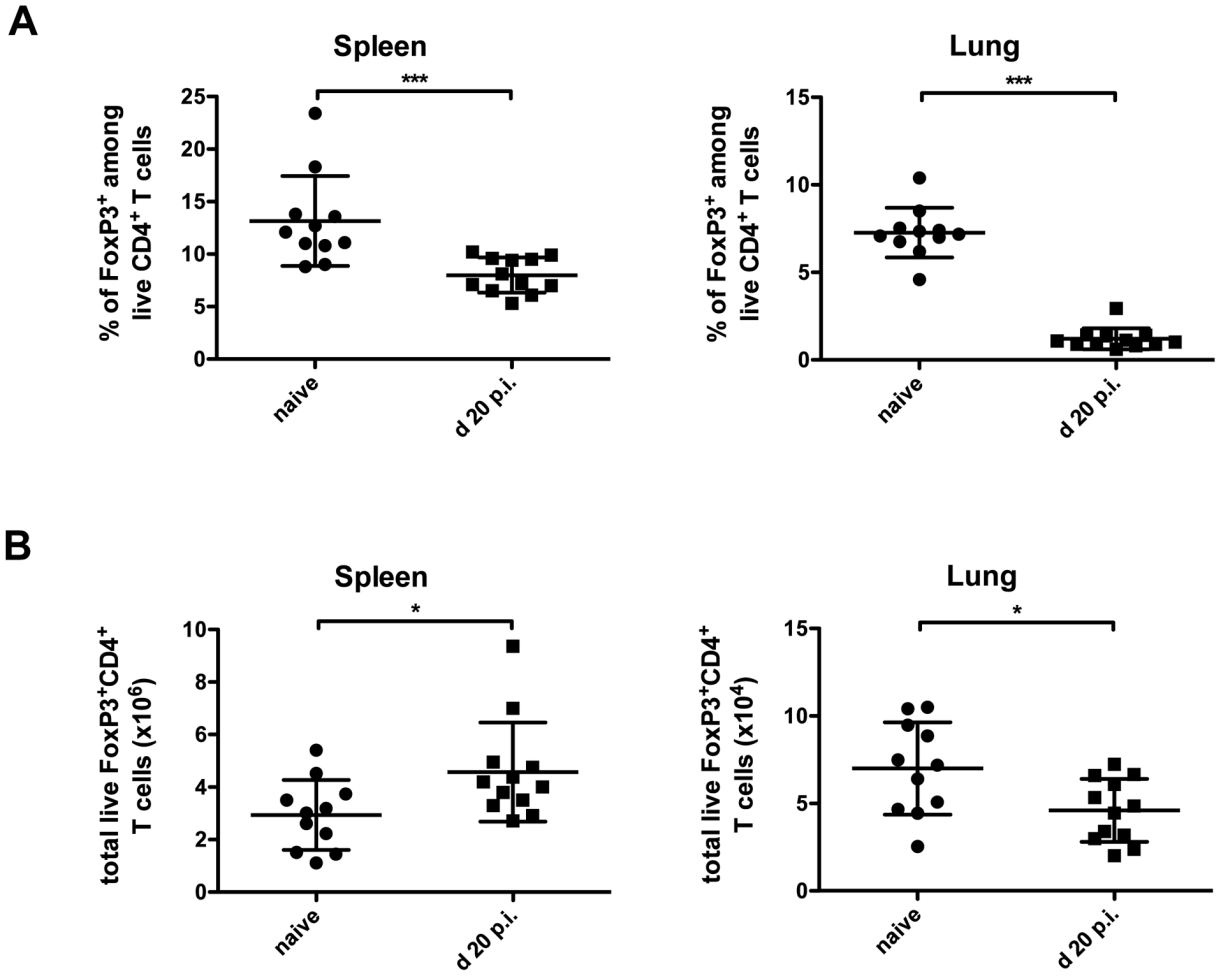
Preferential expansion of Teff cells over Tregs during acute BCG infection. WT mice were infected i.v. with 2×10^6^ CFU *M. bovis* BCG (black squares), or not (black dots), and the (A) frequency and (B) total cell number of FoxP3^+^ Tregs within the live CD4^+^ T cell gate was determined in spleen (left) and lungs (right) at day 20 p.i.. Data are pooled from three independent experiments and represent the mean ± SD of 11–12 mice per group. Each symbol represents an individual mouse. N = 3. Statistical analysis: Mann-Whitney-U-Test. *p<0.05; **p<0.01; and ***p<0.001.

Tregs have been found at high percentages in the blood of Tb infected patients [10] as well as in the lung granulomas and lymphoid organs of *Mtb* infected mice [9]. To check whether this expansion was analogous in *M. bovis* BCG infection, we first analysed the expression of FoxP3 in the CD4^+^ T cell population during the course of infection. As shown in Figure 1A, the percentage of FoxP3^+^CD4^+^ T cells observed at day 20 post infection (p.i.) was reduced in the lungs and spleen of *M. bovis* BCG infected mice compared to non-infected controls. This reduction was only transient as it recovers later during infection (Figure S1A, B, left panels). To determine whether this contraction of the Treg population resulted from a reduction in the total number of FoxP3^+^CD4^+^ T cells or an expansion of effector FoxP3^−^CD4^+^ T cells, we calculated the absolute number of FoxP3^+^CD4^+^ and FoxP3^−^CD4^+^ T cells. At day 20 p.i., the total number of FoxP3^+^CD4^+^ T cells in the lungs of infected mice was slightly reduced compared to non-infected controls (Figure 1B, right panel), but returns later to normal levels (Figure S1A, middle panel). On the contrary, at this time point, the total number of FoxP3^+^CD4^+^ T cells in the spleen of infected mice was higher than in non-infected controls (Figure 1B, left panel) and remained increased at later time points (Figure S1B, middle panel). Thus, our results suggest that during the early phase of mycobacterial infection, a preferential expansion of FoxP3^−^CD4^+^ effector T cells occurs (Figure S1A, B, right panels), leading to a transient contraction in the Treg fraction both in the lungs and spleen.

### Tregs suppressive capacity is not altered upon *M. bovis* BCG infection

**Figure 2:**
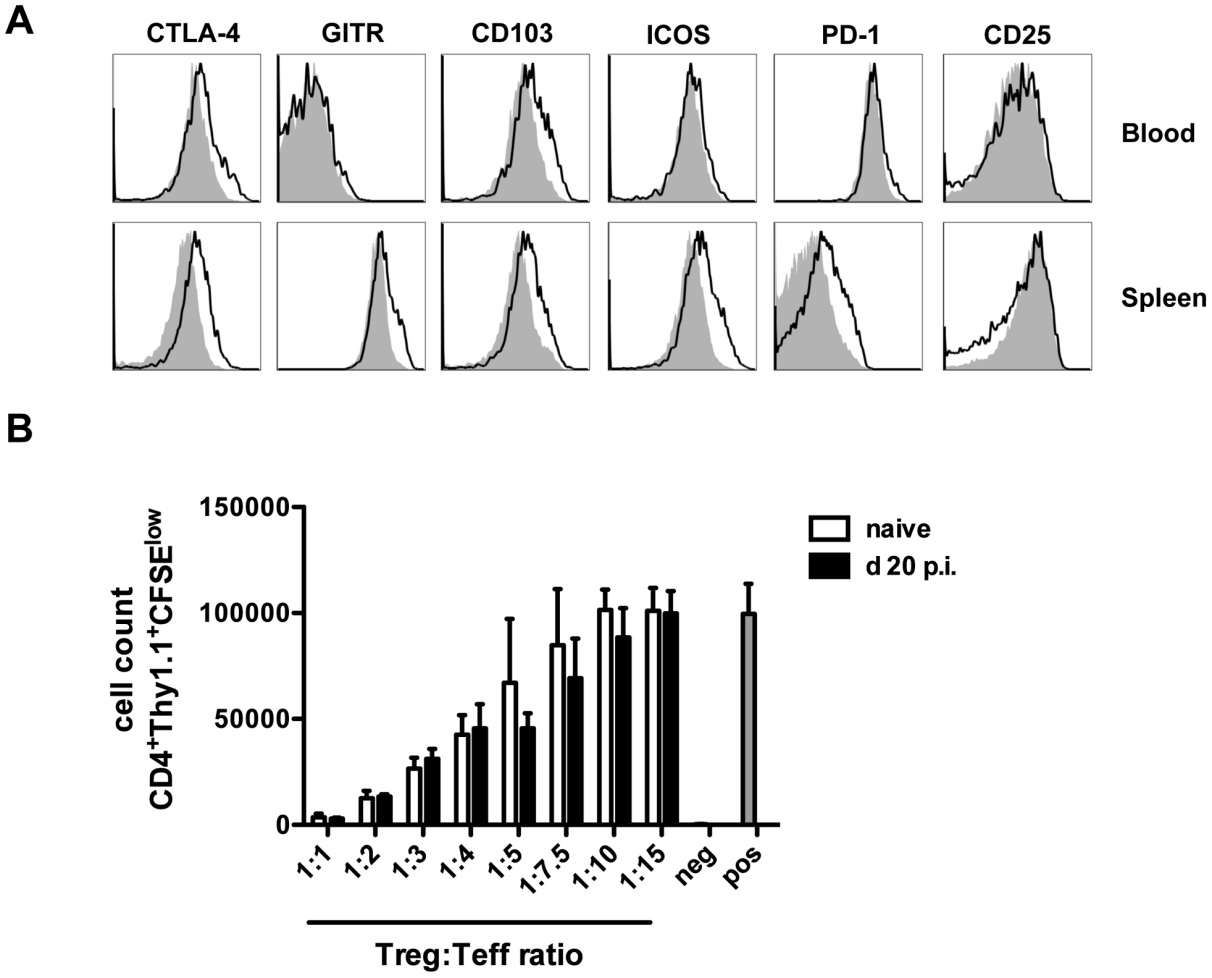
*M. bovis* BCG infection induces Treg activation but does not affect their suppressive capacity. Mice were infected i.v. with 2×10^6^ CFU *M. bovis* BCG, or not, and the phenotype and suppressive capacity of Tregs was evaluated at day 20 p.i. (A) Expression of CTLA-4, GITR, CD103, ICOS, PD-1 and CD25 within the FoxP3^+^CD4^+^ Treg population in the blood (upper panel) and spleen (lower panel) of *M. bovis* BCG-infected (black line) *vs* non-infected (grey shaded area) WT mice. Results show representative FACS plots of 3 mice per group. N = 2. (B) Suppressive capacity of Tregs isolated from naïve (white bars) or day 20 p.i. (black bars) *M. bovis* BCG-infected DEREG mice determined by an *in vitro* suppression assay. Proliferation of CD25^−^CD4^+^ Thy1.1^+^ effector T cells was assessed using different ratios of Treg:Teffector cells in the presence of 1 µg/mL soluble αCD3. For the negative control, αCD3 was omitted. Means of three wells ± SD are depicted. N = 3.

It has been previously reported that subsets of Tregs retain a certain degree of plasticity, being able to become inflammatory cells in response to environmental cues [34]. Therefore, we determined whether Treg function is sustained during mycobacterial infection. As shown in Figure 2A, 20 days after *M. bovis* BCG infection, FoxP3^+^CD4^+^ Treg cells in the blood upregulated characteristic markers of activated Tregs such as CTLA-4 and CD103, but not ICOS, PD-1 or GITR. Moreover, Tregs found in the spleen upregulated all of these markers, but expressed slightly lower levels of CD25 (Figure 2A). We next asked whether Tregs maintain their suppressive function after infection. To this aim, we tested the capacity of FoxP3^+^CD4^+^ T cells to suppress T cell proliferation *in vitro*. eGFP^+^CD4^+^ T cells (Treg cells) were isolated from naïve or *M. bovis* BCG infected DEREG mice 20 days p.i.. Effector T cells were isolated from Thy1.1 non-infected mice. eGFP^+^ Tregs from *M. bovis* BCG infected mice suppressed effector T cell proliferation to the same extent as eGFP^+^ Tregs from naïve DEREG mice (Figure 2B). Taken together, our data suggest that upon mycobacterial infection, Tregs become activated and have a similar capacity to suppress CD4^+^ T cell proliferation as naïve Tregs.

### Treg depletion has only marginal effects on *M. bovis* BCG burden

**Figure 3:**
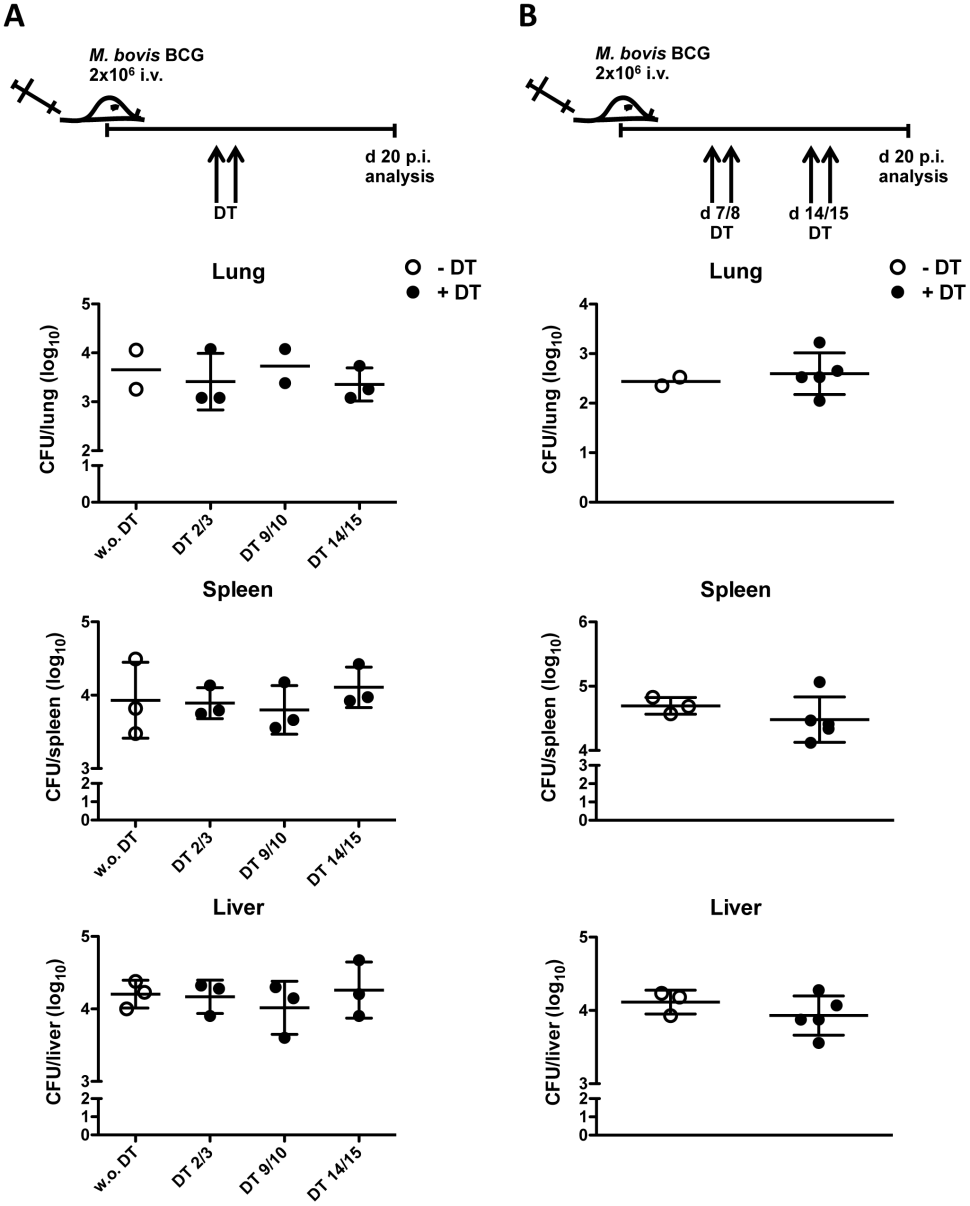
Minor effect of Treg depletion on *M. bovis* BCG bacterial burden. (A) DEREG mice were infected i.v. with 2×10^6^ CFU *M. bovis* BCG and Tregs were depleted by DT administration on days 2/3, 9/10 or 14/15 (black dots) in a single-depletion round or left untreated (white dots). CFU were determined in lungs, spleen and liver 20 days p.i.. Each symbol represents an individual mouse. Data represent mean ± SD of 3 mice per group. N = 1. (B) DEREG mice were infected i.v. with 2×10^6^ CFU *M. bovis* BCG and Tregs were depleted by DT administration on days 7/8 and 14/15 (black dots) in two rounds of depletion or were left untreated (white dots). CFU were assessed in lungs, spleen and liver on day 20 p.i.. Each symbol represents an individual mouse. Data represent mean ± SD of 3–5 mice per group. N = 3.

Having demonstrated the suppressive capacity of Tregs during mycobacterial infection, we speculated that specific Treg depletion might indeed contribute to enhanced immune responses and lower pathogen burden. Previous studies involving Treg depletion during mycobacterial infection have led to contradictory results regarding the contribution of this cell population to bacterial control. In addition, in most of these studies, Treg depletion was performed before or early after infection [9], [15], [21], but expansion of mycobacteria-specific Tregs occurs later during infection [9], [35], [36]. Thus, to determine whether Treg depletion after infection influences bacterial burden, we made use of DEREG mice that allow the specific depletion of FoxP3^+^CD4^+^ Tregs cells by administering DT. Mice were infected intravenously (i.v.) with 2×10^6^ colony forming units (CFU) *M. bovis* BCG and DT was given intraperitoneally (i.p.) on two consecutive days at different time points after infection. CFU were enumerated in the lungs, spleen and liver at day 20 p.i.. Depletion of Tregs during the early phase of mycobacterial infection did not affect bacterial burden as evidenced by the number of CFU detected in the lungs, spleen and liver of DEREG DT-treated mice compared to non-treated DEREG mice or WT DT-treated controls (Figure 3A and data not shown). Since Tregs can rebound and refill their niche after a certain period of time, we next tested a different protocol, by applying two rounds of DT on days 7/8 and 14/15. However, no differences were observed in the bacterial load present in the lungs, spleen and liver of DT-depleted *vs* non-depleted mice at day 20 p.i. (Figure 3B), suggesting that depletion of Tregs at the time when adaptive T cell responses are initiated has no impact on pathogen burden.

Since we observed that Treg expansion occurs after day 20 p.i., we next analysed the effect of Treg depletion on bacterial burden in the later course of infection (Figure S2). To address this, mice were infected with *M. bovis* BCG and DT was given i.p. on days 11/12, 18/19 and 25/26 p.i. (Figure S2A). Bacterial burden was determined on days 20, 28 or 35 p.i.. Still, with the exception of a small reduction in the CFU in spleen and the same tendency in the liver on day 35 p.i., similar bacterial loads were detected in the lungs, spleen and liver of depleted *vs* non-depleted mice (Figure S2B). Collectively, these results point to a limited role of Treg depletion on the control of *M. bovis* BCG infection.

### Treg depletion only partially affects inflammatory cytokine production

**Figure 4:**
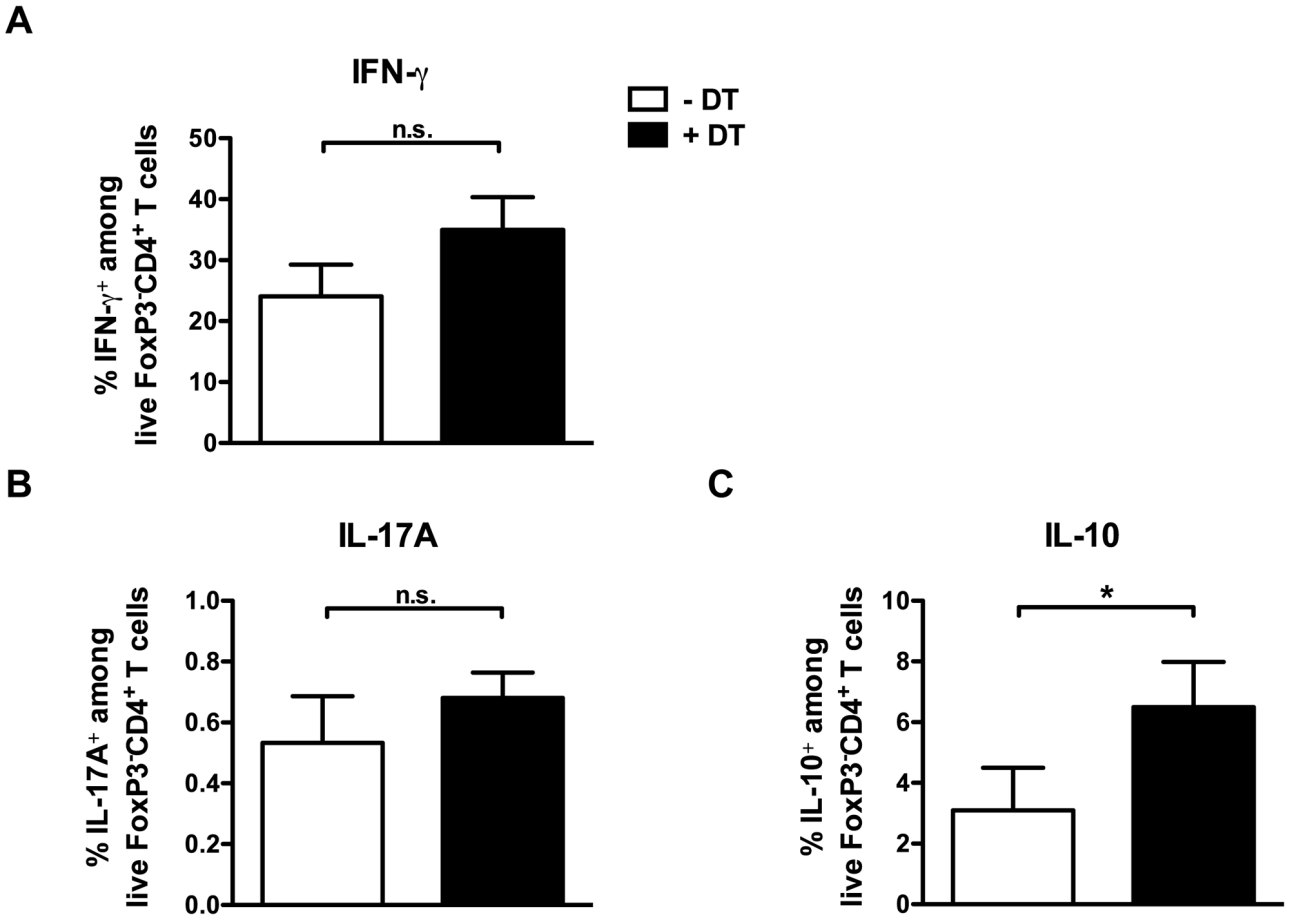
Limited impact of Treg depletion on inflammatory cytokine production after *M. bovis* BCG infection. Intracellular cytokine production by FoxP3^−^CD4^+^ T cells was analysed in the spleen of day 7/8 and 14/15 double-depleted (black bars) or untreated DEREG mice (white bars) on day 20 after i.v. infection with 2×10^6^ CFU *M. bovis* BCG. Percentages of (A) IFN-γ, (B) IL-17A and (C) IL-10 production within live FoxP3^−^CD4^+^ T cells after PMA/ionomycin restimulation are shown. Bar graphs represent mean ± SD of 3–5 mice per group. N = 3. Statistical analysis: Mann-Whitney-U-Test. *p<0.05; **p<0.01; and ***p<0.001.

Removal of Tregs has been associated with an increase in IFN-γ production by T cells, a cytokine that is crucial for protection against mycobacteria [37], [38]. To determine whether this was similar in our model, we measured cytokine production by conventional FoxP3^−^CD4^+^ T cells after Treg depletion in the spleen of DEREG mice. As shown in Figure 4A, depletion of Tregs on days 7/8 and 14/15 after *M. bovis* BCG infection led to a slight but not significant increase in the percentage of IFN-γ-producing FoxP3^−^CD4^+^ T cells on day 20 p.i., whereas no difference was observed for IL-17A production compared to non-depleted mice (Figure 4B). Interestingly, a significantly higher percentage of FoxP3^−^CD4^+^ T cells from DEREG depleted mice produced the anti-inflammatory cytokine IL-10 compared to non-depleted mice (Figure 4C). Taken together, our data suggest that depletion of Tregs after *M. bovis* BCG infection only marginally affects the pro-inflammatory cytokine response against mycobacteria, while promoting IL-10 production by CD4^+^ T cells.

### Rapid rebound of the Treg niche upon depletion limits bacterial control and prevents autoimmunity

**Figure 5:**
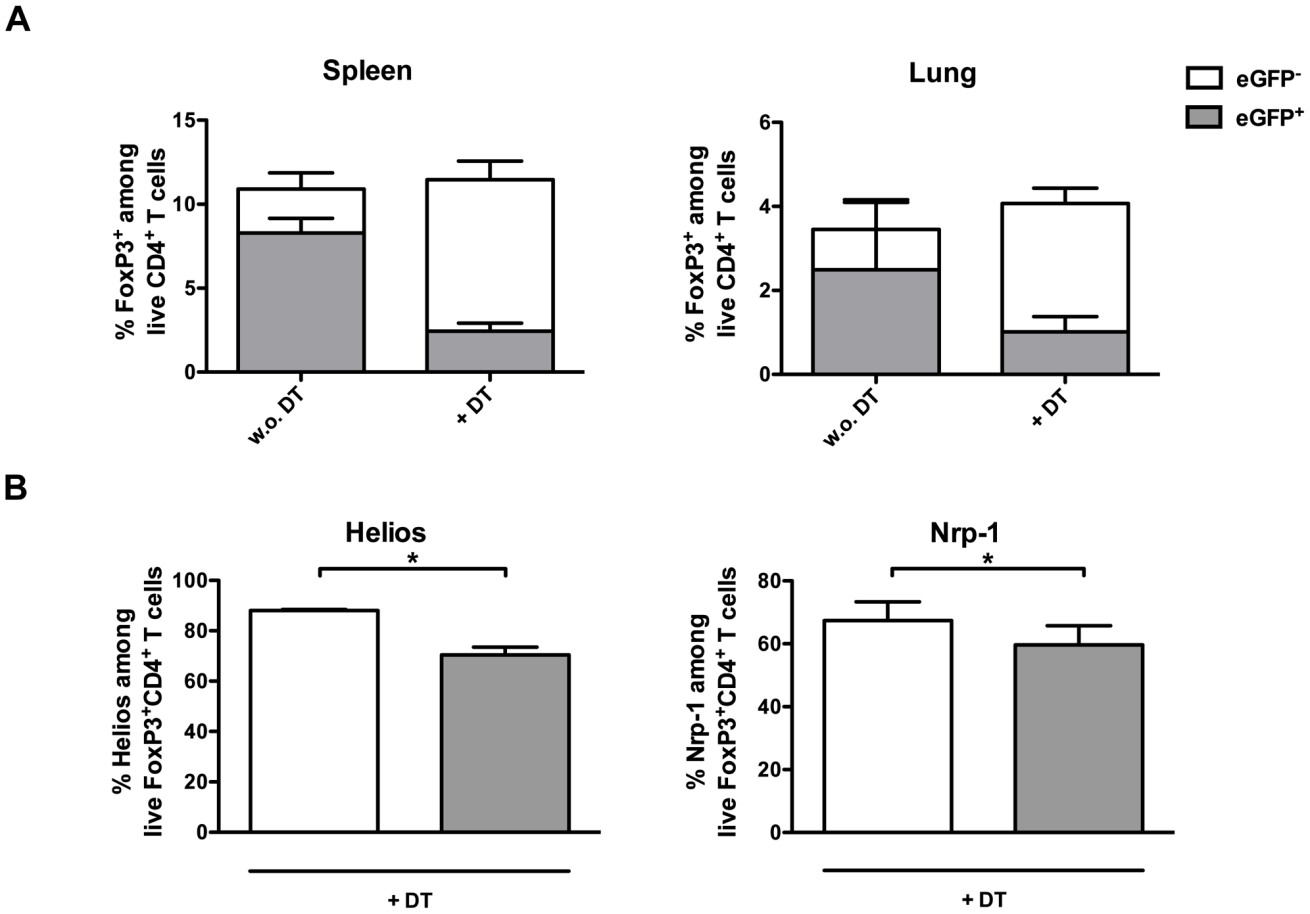
eGFP^−^ diTregs rapidly replenish the pool of Tregs in *M. bovis* BCG infected DEREG mice. DEREG mice were infected i.v. with 2×10^6^ CFU *M. bovis* BCG and treated, or not, with DT on days 7/8 and 14/15 p.i. and (A) the percentage of eGFP^+^ (grey)- and eGFP^−^ (white) Foxp3^+^CD4^+^ Tregs cells in the spleen (left) and lungs (right) or (B) the expression of the thymic Treg markers Helios (left) and Nrp-1 (right) in the eGFP^+^ (grey) and eGFP^−^ (white) Treg population were analysed in the spleen at day 20 p.i. in DT-treated mice. Bar graphs represent mean ± SD of 3–5 mice per group. N = 3. Statistical analysis: Mann-Whitney-U-Test. *p<0.05; **p<0.01; and ***p<0.001.

We previously described a small population of eGFP^−^FoxP3^+^CD4^+^ Tregs that is insensitive to DT and preferentially expands in DEREG mice upon repeated DT treatment [27], [39]. Thus, we next checked whether eGFP^−^FoxP3^+^CD4^+^ DT-resistant Tregs (diTregs) expand after DT treatment in mycobacteria infected mice. As shown in Figure 5, depletion of Tregs during mycobacterial infection in DEREG mice leads to a reduction in the eGFP^+^FoxP3^+^CD4^+^ T cell population both in the spleen and the lungs, but a concomitant increase in eGFP^−^FoxP3^+^CD4^+^ diTregs (Figure 5A). To analyse whether diTregs represent a separate subset of Tregs or only differ in the eGFP expression, we analysed expression of Neuropilin-1 (Nrp-1) and Helios, both markers of thymus-derived Tregs [40], [41] in DT-treated mice. Slightly higher frequencies of Nrp-1 and Helios positive cells were found in the eGFP^−^FoxP3^+^CD4^+^ population compared to eGFP^+^FoxP3^+^CD4^+^ T cells (Figure 5B).

To further characterise the importance of diTregs in limiting bacterial control upon Treg depletion, we made use of DEREG mice crossed to FoxP3^GFP^ mice (D× FoxP3^GFP^), a knock-in strain that expresses GFP fused at the N-terminal of FoxP3 [42]. Treg homeostasis in these commonly used FoxP3^GFP^ reporter mice is impaired due to altered interactions of the Foxp3-GFP fusion protein with several transcription factors [43], [44]. D× FoxP3^GFP^ mice were infected with 2×10^6^ *M. bovis* BCG i.v. and Treg depletion was performed in two rounds (Figure S3A). On day 20 p.i., both homozygous and heterozygous DT-treated D× FoxP3^GFP^ mice exhibited decreased bacterial burden in the lungs, liver and spleen compared to non-treated D× FoxP3^GFP^ mice (Figure S3B). Moreover, D× FoxP3^GFP^ mice showed lower CFU in the spleen than DEREG mice upon Treg depletion. This effect was not mediated by lower susceptibility of D× FoxP3^GFP^ mice towards *M. bovis* BCG infection in general since untreated D× FoxP3^GFP^ and DEREG mice displayed no differences in the bacterial load. Importantly, the capacity of Tregs to refill the Treg compartment upon DT treatment was impaired in D× FoxP3^GFP^ mice as evidenced by the lack of FoxP3 expressing cells, particularly in homozygous mice (Figure S3C). In turn, a significantly higher frequency of IFN-γ-producing CD4^+^ T cells was measured in DT-treated D× FoxP3^GFP^ compared to DT-treated DEREG mice or mice that were not treated with DT (Figure S3D). However, DT-treated D× FoxP3^GFP^ also developed characteristic signs of autoimmunity such as blepharitis and multi-organ pathology (data not shown). Careful characterisation of these mice indicates that autoimmune disease is independent of the infection and is featured by massive T cell activation, myeloproliferation and the production of autoantibodies (Mayer *et al*., unpublished data). Together, these data indicate that only in situations in which Treg homeostasis is abolished, Treg depletion can impact mycobacterial burden.

### Treg depletion does not influence bacterial burden during *Mtb* infection

**Figure 6:**
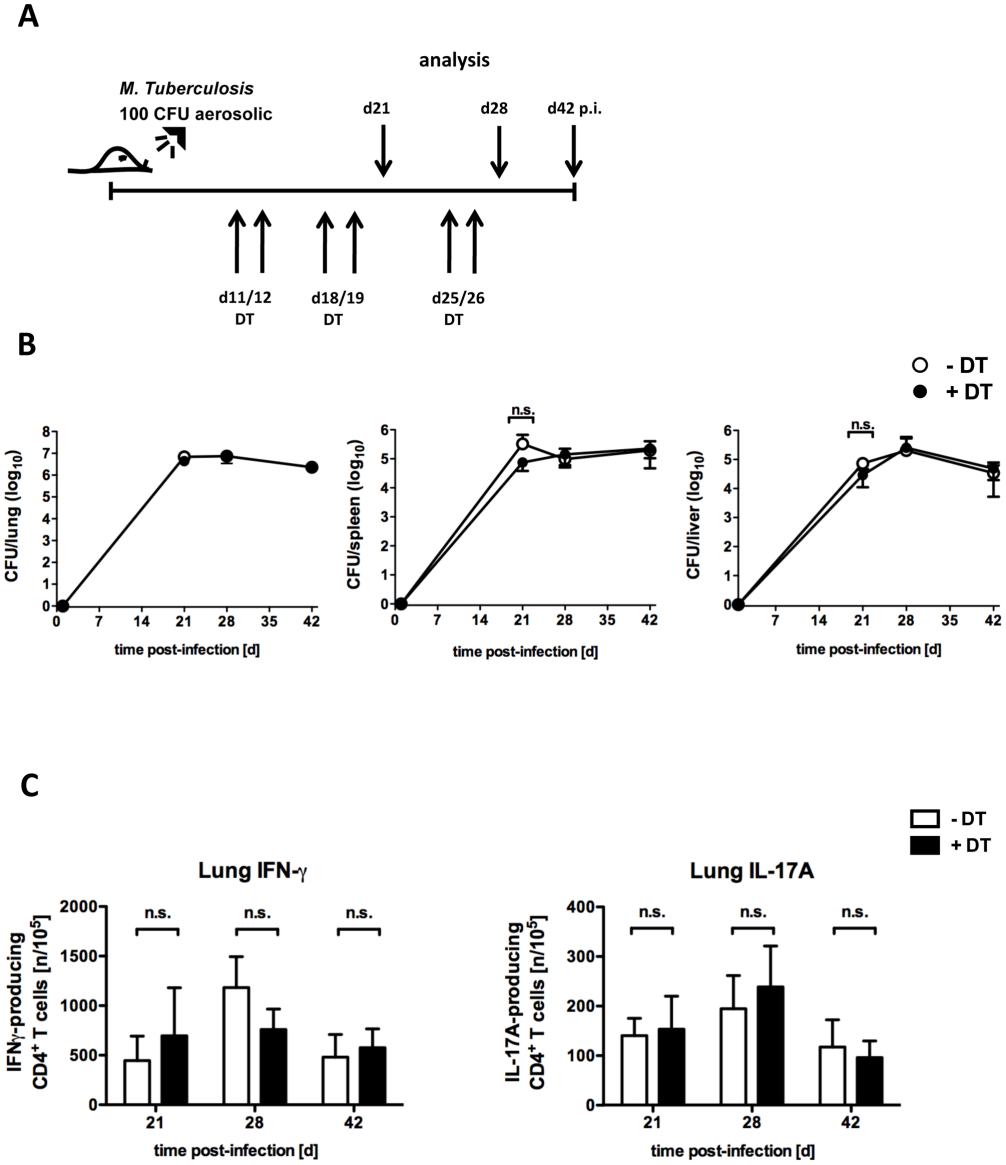
Removal of Tregs after *Mtb* aerosol infection in DEREG mice does not influence pathogen control. DEREG mice were infected with approx. 100*Mtb* via the aerosol route. Tregs were depleted by DT administration on days 11/12, 18/19 and 25/26 (black dots/bars) or not (white dots/bars). (A) Experimental scheme. (B) Mycobacterial colony enumeration assays were performed in lungs (left), spleen (middle) and liver (right) on day 20, 28 and 42 p.i.. Data represent mean ± SD of 5 mice per group. (C) The frequency of ESAT6_1–20_-specific IFN-γ- and IL-17A-producing CD4^+^ cells per 10^5^ total lung cells was determined by ELISPOT assay at different time points after infection. Bar graphs represent the mean ± SD of 5 mice per group. N = 3 (day 20), N = 1(day 28 and 42).

A previous study suggests that the removal of Tregs during *Mtb* aerosol infection leads to reduced bacterial burden in the lung, but not in the spleen of infected mice [9]. In this model however, repopulation of the Treg niche was impaired and therefore, mice developed signs of autoimmune activation. Thus, we next examined whether depletion of Tregs during the course of *Mtb* aerosol infection also affects bacterial control in DEREG mice that do not develop autoimmunity because of temporary Treg depletion. To this aim, bacterial loads were determined in lungs, spleen and liver of untreated and DT-treated DEREG mice infected with 100 CFU *Mtb* via the aerosol route (Figure 6A). Similar to the *M. bovis* BCG infection model, depletion of Tregs did not influence bacterial burden in any of the organs investigated, even at later time points during infection (Figure 6B). To further analyse the impact of Treg depletion on the differentiation of antigen-specific IFN-γ- and IL-17A-producing CD4^+^ T cells during experimental Tb, lung cells were isolated at different time points after aerosol *Mtb* infection, restimulated with ESAT6_1–20_-pulsed antigen-presenting cells and the frequency of cytokine-producing cells was determined by ELISPOT. The relative amount of IFN-γ- and IL-17A-secreting CD4^+^ T cells in the lungs was comparable between DT-treated mice and untreated littermates (Figure 6C). Of note, in both mycobacterial infection models the production of IL-17A was only marginal compared to IFN-γ production. Together, these data suggest that under physiologic conditions in which Tregs can repopulate their niche, the removal of Tregs does not improve mycobacterial control.

## Discussion

The development of new strategies to specifically target Tregs has raised the question of whether this approach can also be applied therapeutically to treat chronic infections. In this sense, the use of mouse models that allow specific depletion of Tregs, such as the DEREG mouse, represents the best possible option to evaluate the feasibility of this strategy *in vivo*. Using this model, here we show that therapeutic depletion of Tregs has only minor effects on *M. bovis* BCG infection and it does not affect *Mtb* control. We demonstrate that the removal of Tregs after mycobacterial infection only marginally affects inflammatory cytokine production, though it promotes IL-10. Moreover, we postulate that the rapid rebound of Tregs that occurs in mycobacteria-infected DEREG mice at the expense of a small population of DT-resistant FoxP3 cells may hamper the impact of Treg depletion on pathogen burden, while preventing autoimmunity.

Our findings and those from others show that mycobacterial infection induces an increase in the total number of Tregs that succeeds the expansion of effector T cells [9], [14], [15]. However, it is not clear whether Treg expansion is an immune evasion mechanism directly exploited by the pathogen to avoid immunity or whether it might represent homeostatic growth to prevent excessive immune responses. Moreover, it is not well understood whether Tregs are functionally suppressive during mycobacterial infection. Here we show that, analogous to *Mtb*, *M. bovis* BCG infection results in an upregulation of different Treg activation markers such as CTLA-4, CD103, ICOS, PD-1 and GITR, all characteristic of highly suppressive “inflammation-seeking Tregs” [45]–[50]. Along the same line, using tetramer-based staining for the ESAT-6 antigen of *Mtb*, Shafiani *et al*. demonstrated that the majority of these activated Tregs is represented by mycobacteria-specific Tregs, suggesting that Treg activation is TCR-dependent [36]. Furthermore, our results show for the first time that during acute mycobacterial infection, the suppressive capacity of Tregs is not disrupted, excluding this as an explanation for the poor ability of Treg depletion to enhance mycobacterial control.

Depletion of Tregs by administration of anti-CD25 antibodies prior to mycobacterial infection has been associated with an increase in the frequency of IFN-γ-producing CD4^+^ effector T cells [15], [21], a mechanism that is crucial for mycobacterial containment [51]. In contrast, our results show that Treg depletion after *Mtb* or *M. bovis* BCG infection only leads to a marginal increase in Th1 responses. However, removal of Tregs during *M. bovis* BCG infection causes an increase in the capacity of both FoxP3^+^ and FoxP3^−^CD4^+^ T cells to produce IL-10, an anti-inflammatory cytokine that inhibits the production of Th1-inducing cytokines such as IL-12 [52]. Likewise, increased susceptibility to BCG [53] and *Mtb* [54] in transgenic mice overexpressing IL-10 in T cells is associated with an impaired Th1 response and IL-10 has been shown to suppress immune responses in Tb patients [55]–[57]. Thus, considering the anti-inflammatory effects of IL-10 signalling on anti-mycobacterial immune responses, it seems reasonable that the elevated IL-10 production observed in DEREG mice after DT-treatment may diminish the impact of Treg depletion on bacterial burden.

Another factor that may contribute to the poor effect of Treg depletion on bacterial control in our model might be the expansion of diTregs. diTregs are a population of DT-resistant Tregs that are indistinguishable from other Tregs and expand after chronic DT administration [27,39 and Mayer *et al*., unpublished data]. Here we observed that upon repeated DT treatment, diTregs can completely replenish the Treg compartment during mycobacterial infection after 5–6 days. Consistent with these results, using bone marrow chimeras in which Tregs can be continuously depleted by means of anti-Thy1.1 antibodies, Scott-Browne *et al*. demonstrated that even a small remaining population of Thy1.1 cells can repopulate the Treg niche when antibody treatment stops [9]. Homeostatic expansion of Tregs after Treg removal can also occur independently of inflammatory conditions, and is believed to be the result of an IL-2-dependent *quorum-sensing* mechanism that ensures a continuous balance in the ratio of T effector to T suppressor cells [58]. Indeed, Scott-Browne's study suggests that permanent Treg depletion can be effective at reducing bacterial burden in the lungs (although not in the spleen), but with a concomitant increase in autoreactive T cells [9]. Along the same line, our results show that disruption of the homeostatic Treg rebound mechanism using DEREG mice crossed to FoxP3^GFP^ knock-in mice [42] improves mycobacterial control, but is accompanied by a strong autoimmune reaction characterised by blepharitis and the presence of highly activated T cells as well as a myeloproliferative disorder (Mayer *et al*., unpublished data). Although the depletion efficiency in DEREG mice and D× FoxP3^GFP^ is theoretically identical (provided by the DT receptor from the BAC), in FoxP3^GFP^ mice, the N-terminus of FoxP3 is tagged with GFP causing altered molecular interactions of FoxP3 with several transcription factors [43], [44]. As a consequence, diTregs in D× FoxP3^GFP^ may have a decreased ability to replenish the Treg niche. Thus, although the study by Scott-Browne and our results with D× Foxp3^GFP^ mice support the current paradigm that Tregs contribute to mycobacterial persistence (although to different degrees), these models are far from being physiological. This raises the question of whether the decreased bacterial burden observed in both models is an indirect effect of the strong immune activation observed in these mice rather than a consequence of the specific depletion of mycobacteria-specific Tregs.

In summary, our results and those from others suggest that under physiological conditions, depletion of Tregs leads to a rapid replenishment of the Treg compartment that may prevent efficient pathogen containment. On the contrary, complete Treg depletion may facilitate mycobacterial control, but carries the risk of autoimmunity. Taking this into account, it is clear that new strategies that allow manipulation of pathogen-specific Tregs without affecting the complete Treg pool are urgently required. In this sense, a recent study by Shafiani *et al*. identified a population of Tregs that recognises a *Mtb*-derived peptide [36]. Targeting this population of Tregs might therefore represent a better option to promote pathogen contention without the disadvantage of autoimmune side effects. A different strategy is the use of antibodies that target surface markers of activated Tregs. Here we show that upon infection, Tregs upregulate CTLA-4, CD103, ICOS, PD-1 and GITR. Although none of these markers is specifically expressed on Tregs and some of them are also present on activated T cells, recent studies demonstrate the efficacy of anti-CTLA-4 or anti-CCR4 to reactivate immune responses against tumours [59], [60]. Hence, it remains to be tested whether these approaches are also useful during chronic mycobacterial infection.

## Material and Methods

### Ethics statement

All animal experiments were performed in compliance with the German animal protection law (TierSchG BGBl. I S. 1105; 25.05.1998). The mice were housed and handled in accordance with good animal practice as defined by FELASA and the national animal welfare body GV-SOLAS. All animal experiments were approved by the Lower Saxony Committee on the Ethics of Animal Experiments as well as the responsible state office (Lower Saxony State Office of Consumer Protection and Food Safety) under the permit numbers 33.12-42502-04-10/0075 and 33.9-42502-04-12/0732 or the Animal Research Ethics Board of the Ministry of Environment (Kiel, Germany- Permit number: V311-7224.123.3 (82–6/12) considering the German Animal Welfare Act. All surgery was performed after mice were euthanised by CO2 and cervical dislocation and all efforts were made to minimise suffering.

### Mice

All animals were bred and maintained under specific pathogen-free conditions at the animal facility of the Helmholtz Centre for Infection Research (HZI, Braunschweig, Germany) or the TWINCORE, Centre for Experimental and Clinical Infection Research (Hannover, Germany). DEREG mice [26] and Thy1.1 mice were both on C57BL/6 background. DEREG mice (on BALB/c background) were crossed to FoxP3^GFP^ mice [42], kindly provided by Ludger Klein (Ludwig-Maximilians University Munich, Germany) and used as heterozygous (D× FoxP3^WT/GFP^) or homozygous mice (D× FoxP3^GFP/GFP^) mice for the FoxP3^GFP^ knock-in gene. For *Mtb* infection experiments, DEREG mice were kindly provided by Thomas Jacobs (Bernhard Nocht Institute for Tropical Medicine, Hamburg, Germany) and kept under barrier conditions at the BSL 3 facility from the Research Center Borstel (Borstel, Germany) in individually ventilated cages. Sex- and age-matched mice between 8- and 16-weeks of age were used in all experiments. WT littermates or non DT-treated DEREG mice served as controls in Treg-depletion experiments.

### Treg depletion

Tregs were depleted at different time points during the experiments by administration of 25 ng/g DT i.p. (Merck/Calbiochem) per mouse on two consecutive days as indicated in the figure legends. Aliquots of DT were prepared and stored at −20°C.

### *M. bovis* BCG infection

For BCG infection experiments, *M. bovis* BCG Pasteur strain was grown at 37°C in Middlebrook 7H9 broth (BD Biosciences) supplemented with 10% Middlebrook OADC enrichment medium (BD Biosciences), 0.002% glycerol (Roth), and 0.05% Tween 80 (Roth). Midlog phase cultures were harvested, aliquoted, and frozen at −80°C. Bacteria were prepared from frozen stocks by thawing at 37°C, resuspension in PBS supplemented with 0.025% Tween 80 (PBS-T), and passaged through a 27G needle. Mice were infected with 2×10^6^ CFU *M. bovis* BCG Pasteur i.v. diluted in PBS-T.

### *Mtb* aerosol infection

For *Mtb* infection experiments, *Mtb* H37rv was grown in Middlebrook 7H9 broth supplemented with 10% Middlebrook OADC enrichment medium, 0.002% glycerol, and 0.05% Tween 80. Midlog phase cultures were harvested, aliquoted, and frozen at −80°C. After thawing, viable cell counts were determined by plating serial dilutions of the cultures on Middlebrook 7H10 agar plates followed by incubation at 37°C. Before infection of experimental animals, stock solutions of *Mtb* were diluted in sterile distilled water and pulmonary infection was performed using an inhalation exposure system (Glas-Col). To infect mice with a low dose of 100 CFU, animals were exposed for 40 min to an aerosol generated by nebulising approximately 5.5 ml of a suspension containing 10^7^ live bacteria. Twenty four hours later, the dose of infection was determined in the lungs of infected mice.

### Flow cytometry and cell sorting

The following antibodies and reagents were purchased from eBioscience: αCD4 (RM4-5 and GK1.5), αCD25 (PC61.5), αThy1.1 (HIS51), αCD103 (2E7), αFoxP3 (FJK-16s), αGITR (DTA-1), αIFN-γ (XMG1.2), αIL-10 (JES5-16E3), αIL-17A (eBio17B7) and Brefeldin A. α CTLA-4 (UC10-4B9) and αHelios (22F6) were purchased from Biolegend and αNrp-1 (polyclonal) from R&D. To analyse intracellular cytokine production, 1×10^7^ spleen cells were restimulated in complete RPMI medium (10% FCS, 10 mM Hepes (GIBCO), 50 µM β-mercaptoethanol (GIBCO), 100 U/ml penicillin/streptomycin (Biochrom) for 4 h with 100 ng/mL phorbol myristate acetate (PMA) and 1 µg/mL ionomycin at 37°C, while 5 µg/ml Brefeldin A (eBioscience) was supplemented for the last 2 h. Dead cells were excluded by Aqua fluorescent reactive dye (Invitrogen) staining. Intranuclear FoxP3 staining as well as co-staining with intracellular cytokines was performed using the Fixation/permeabilisation kit (eBioscience) according to manufacturer's instructions. Intracellular cytokine staining without FoxP3 staining was performed using 0.5% saponin after paraformaldehyde-fixation. Data acquisition was performed on a LSRII (BD) or a CyAn ADP (Beckman Coulter) flow cytometer. Cell sorting was conducted at the Cell Sorting Core Facility of the Hannover Medical School using FACSAria (BD), XDP or MoFlo (both Beckman Coulter) machines. Subsequent data analysis was performed with FlowJo software (Tree Star).

### *In vitro* suppression assay

Bone marrow-derived dendritic cells (BMDC) were used as antigen presenting cells. Therefore, BM cells were prepared from femurs and tibiae of Thy1.2^+^ WT mice, and cultured for 7–8 days in complete RPMI medium supplemented with 5% culture supernatant of a granulocyte-macrophage-colony-stimulating factor (GM-CSF)-producing cell line [61]. Naïve CD25^−^CD4^+^ T effector cells were enriched from spleen and lymph nodes of Thy1.1^+^ mice by negative magnetic sorting using the Dynal Mouse CD4 negative T cell isolation kit (Invitrogen) and the CD25 MicroBead Kit (Miltenyi Biotec) following the manufacturer's protocol. FoxP3^+^CD4^+^ Tregs were isolated by FACS sorting of eGFP^+^CD4^+^ cells obtained from spleen and lymph nodes of *M. bovis* BCG infected or naive DEREG mice. Cultures were established in 96-well round bottom plates (Cellstar) in 200 µL complete RPMI medium using APC and different ratios of Treg:Teffector cells. Cultures were stimulated using 1 µg/mL of soluble αCD3 monoclonal antibody (clone 145-2C11, BioXCell). After 5 days, proliferation of CFSE (Life Technologies) -labelled T effector cells was determined by flow cytometry.

### Colony enumeration assay

To determine colony forming units (CFU), BCG infected mice were killed at day 20 and lungs, livers and spleens were aseptically removed. Livers were collected in sterile bags (Nasco) containing 1 mL WTA lysis buffer (0.01% Tween-80 and 0.05% BSA (Roth) in dH_2_0) and mechanically disrupted. Single cell suspensions were prepared from lungs and spleens. Aliquots were taken and diluted in WTA lysis buffer. Viable cell counts were ascertained by plating serial dilutions of organ homogenates on Middlebrook 7H11 agar plates (BD Biosciences) supplemented with 10% OADC and 0.5% glycerol. Plates were incubated at 37°C and CFU were enumerated after incubation at 37°C for 3 weeks. Similarly, after *Mtb* infection, bacterial loads in lungs, spleens and livers were evaluated by enumerating CFU. Therefore, organs from sacrificed animals were removed aseptically, weighed and homogenised in PBS containing a proteinase inhibitor cocktail (Roche Diagnostics) prepared according to the manufacturer's instructions. Tenfold-serial dilutions of organ homogenates were plated in duplicates onto Middlebrook 7H10 agar plates containing 10% OADC and colonies enumerated after incubation at 37°C for 3 weeks. Data are presented as log_10_ CFU per organ.

### ESAT6_1–20_-specific ELISPOT assays

For measuring the frequency of antigen-specific CD4^+^ T cells after *Mtb* infection, single cell suspensions from lungs were prepared and collected in Iscoves-modified Dulbeccos medium (IMDM; Life Technologies) supplemented with 10% FCS (Life Technologies), 0.05 mM β-mercaptoethanol (Sigma), and penicillin and streptomycin (100 U/ml and 100 µg/ml; Life Technologies). Lung cells were restimulated for 20 h with inactivated spleen cells from uninfected mice that have been pulsed with the MHC class II peptide ESAT-6_1–20_ (Research Center Borstel). Detection of antigen-specific IFN-γ- and IL-17A-producing CD4^+^ T cells was conducted using ELISPOT assay kits as described by the manufacturer (BD Bioscience and R&D Systems, respectively). Spots were automatically enumerated using an ELISPOT reader (EliSpot 04 XL; AID) and the frequency of cytokine-producing cells was determined.

### Statistical analysis

Data analyses were performed using GraphPad Prism Software version 5.0 (GraphPad Software) and statistics were calculated using Mann-Whitney-U-Test or Tukey's multiple comparison test. *P-values* were considered significant as follows: *p<0.05; **p< 0.01; and ***p<0.001.

## Supporting Information

Figure S1 **Expansion of FoxP3^−^CD4^+^ T effector and FoxP3^+^CD4^+^ Treg cells during BCG infection.** WT mice were infected i.v. with 2×10^6^ CFU *M. bovis* BCG or not, and the frequency (left) and total cell number of FoxP3^+^ (middle) and FoxP3^−^ (right) cells within the live CD4^+^ T cell gate was determined in the lungs (A) and spleen (B) at different time points after infection (day 7–34 p.i.). Each symbol represents an individual mouse. N = 1.
(TIF)

Figure S2 **Marginal effect of long-term Treg depletion on bacterial burden.** DEREG mice were infected i.v. with 2×10^6^ CFU *M. bovis* BCG. Tregs were depleted by DT administration on days 11/12, 18/19 and 25/26 (black dots) or not (white dots). (A) Experimental schema. (B) CFU were determined in lungs, spleen and liver on day 20 (upper panel), 28 (middle panel) and 35 (lower panel) p.i.. Each symbol displays an individual mouse. Data represent mean ± SD of 4–5 mice per group. N = 1. Statistical analysis: Mann-Whitney-U-Test. *p<0.05; **p< 0.01; and ***p<0.001.
(TIF)

Figure S3 **Impaired rebound of Tregs in D× FoxP3^GFP^ mice results in reduced bacterial burden, but is associated with autoimmunity.** DEREG mice were infected i.v. with 2×10^6^ CFU *M. bovis* BCG. Tregs were depleted by DT administration on days 7/8 and 14/15 (black symbols) or not (white symbols). (A) Experimental schema. (B) CFU were assessed in lungs, spleen and liver on day 20 p.i.. (C) Representative FACS-plots of FoxP3- and GFP-expression by CD4^+^ T cells from DEREG, D× FoxP3^WT/GFP^ and D× FoxP3^GFP/GFP^ mice on day 20 p.i. treated with two rounds of DT or non-treated. (D) Frequencies of intracellular IFN-γ production by live FoxP3^−^CD4^+^ T cells in the spleen of DT-treated or untreated DEREG (D, dots), D× FoxP3^WT/GFP^ (squares) and D× FoxP3^GFP/GFP^ (triangles) mice. (B,D) Each symbol shows an individual mouse. Data represent mean ± SD of 2–5 mice per group. N = 1. Statistical analysis: Mann-Whitney-U-Test. *p<0.05; **p<0.01; and ***p<0.001.
(TIF)
