# Supplementary figures and images for: Rapid Rebound of the Treg Compartment in DEREG Mice Limits the Impact of Treg Depletion on Mycobacterial Burden, but Prevents Autoimmunity
Source: PLoS One. 2014 Jul 22;9(7):e102804. doi: 10.1371/journal.pone.0102804 (PMC4106855; doi:10.1371/journal.pone.0102804)

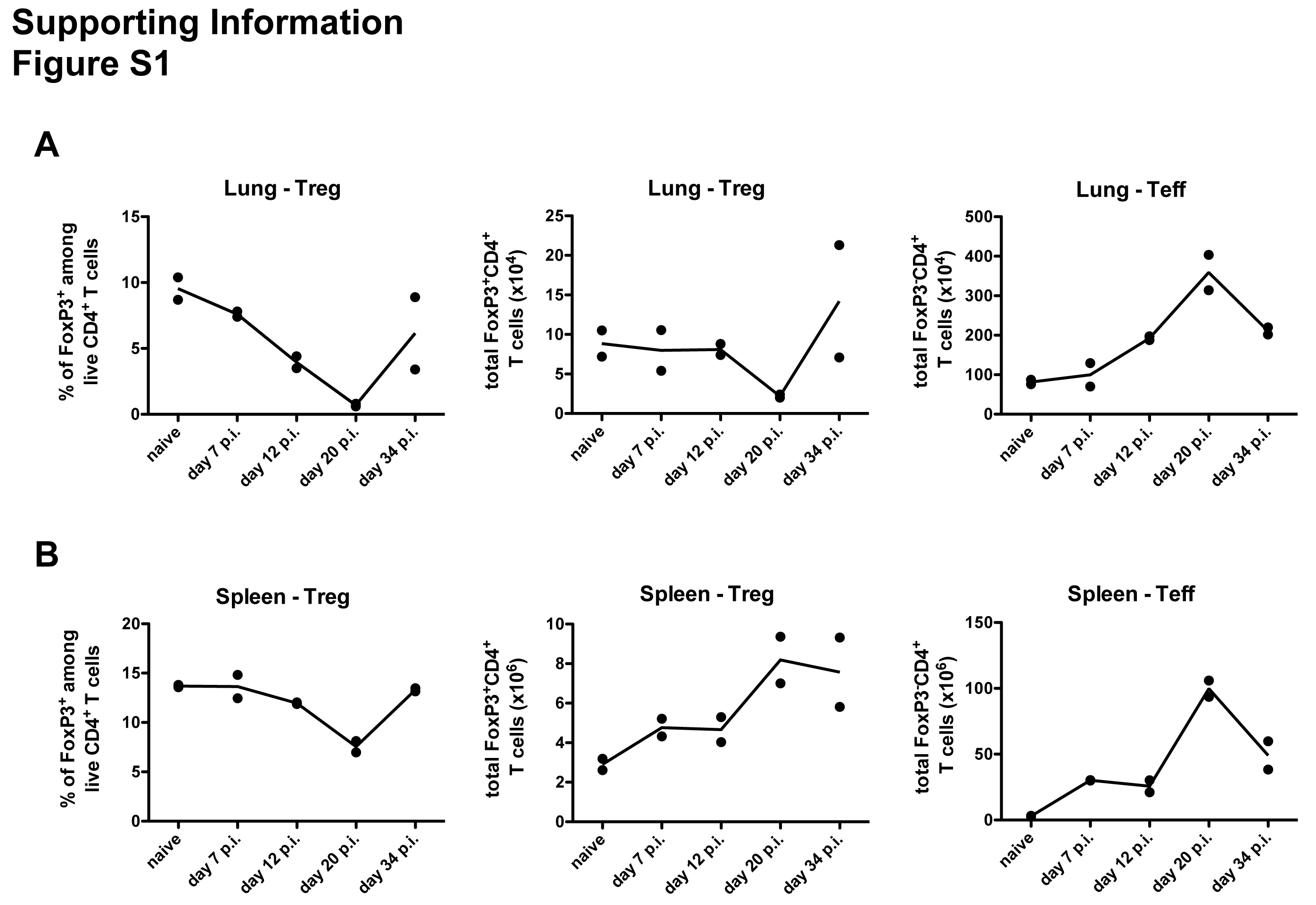

Supplement: Figure S1 — Expansion of FoxP3−CD4+ T effector and FoxP3+CD4+ Treg cells during BCG infection. WT mice were infected i.v. with 2×10^6^ CFU M. bovis BCG or not, and the frequency (left) and total cell number of FoxP3+ (middle) and FoxP3− (right) cells within the live CD4+ T cell gate was determined in the lungs (A) and spleen (B) at different time points after infection (day 7–34 p.i.). Each symbol represents an individual mouse. N = 1. (TIF) [file pone.0102804.s001.tif]

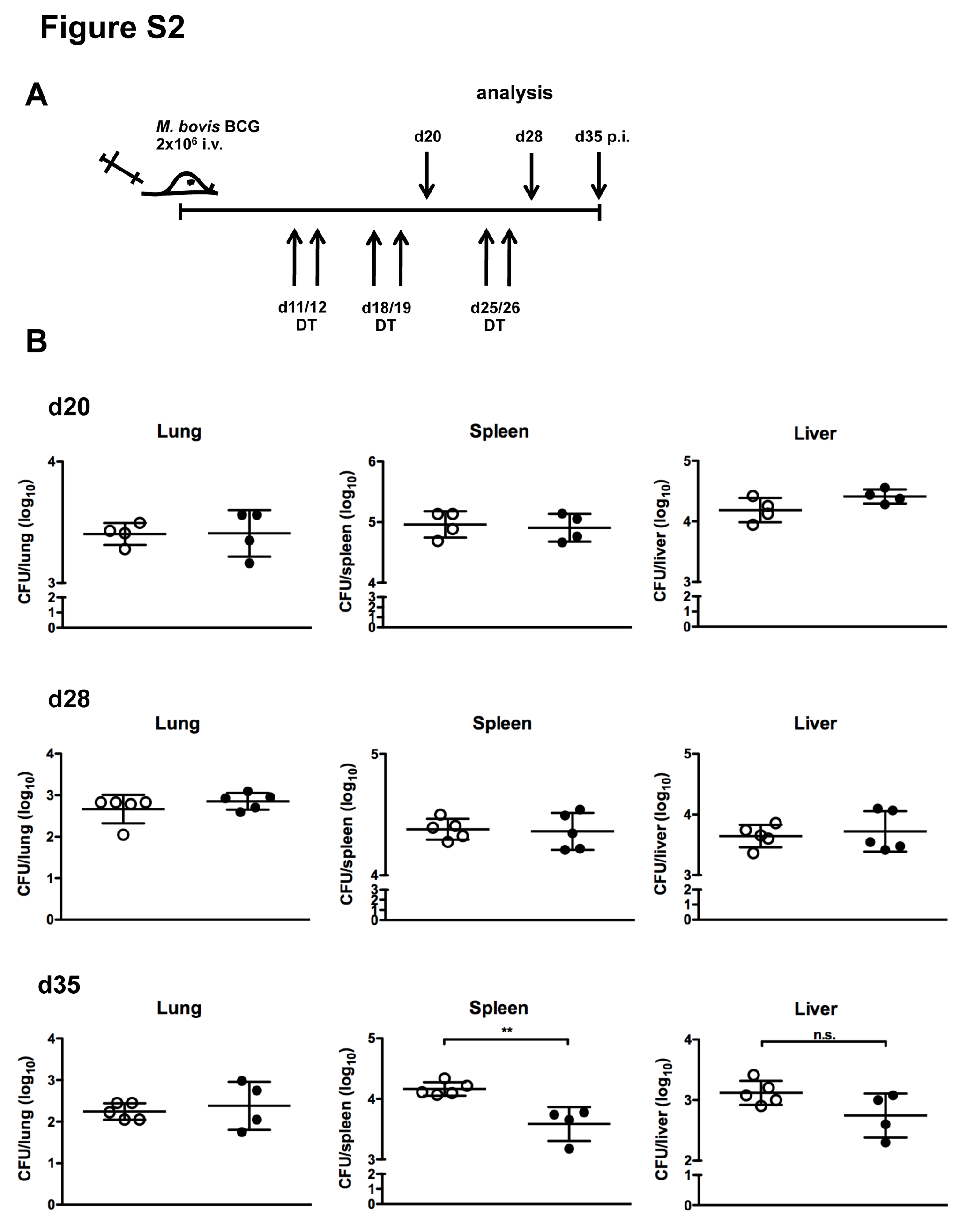

Supplement: Figure S2 — Marginal effect of long-term Treg depletion on bacterial burden. DEREG mice were infected i.v. with 2×10^6^ CFU M. bovis BCG. Tregs were depleted by DT administration on days 11/12, 18/19 and 25/26 (black dots) or not (white dots). (A) Experimental schema. (B) CFU were determined in lungs, spleen and liver on day 20 (upper panel), 28 (middle panel) and 35 (lower panel) p.i.. Each symbol displays an individual mouse. Data represent mean ± SD of 4–5 mice per group. N = 1. Statistical analysis: Mann-Whitney-U-Test. *p<0.05; **p< 0.01; and ***p<0.001. (TIF) [file pone.0102804.s002.tif]

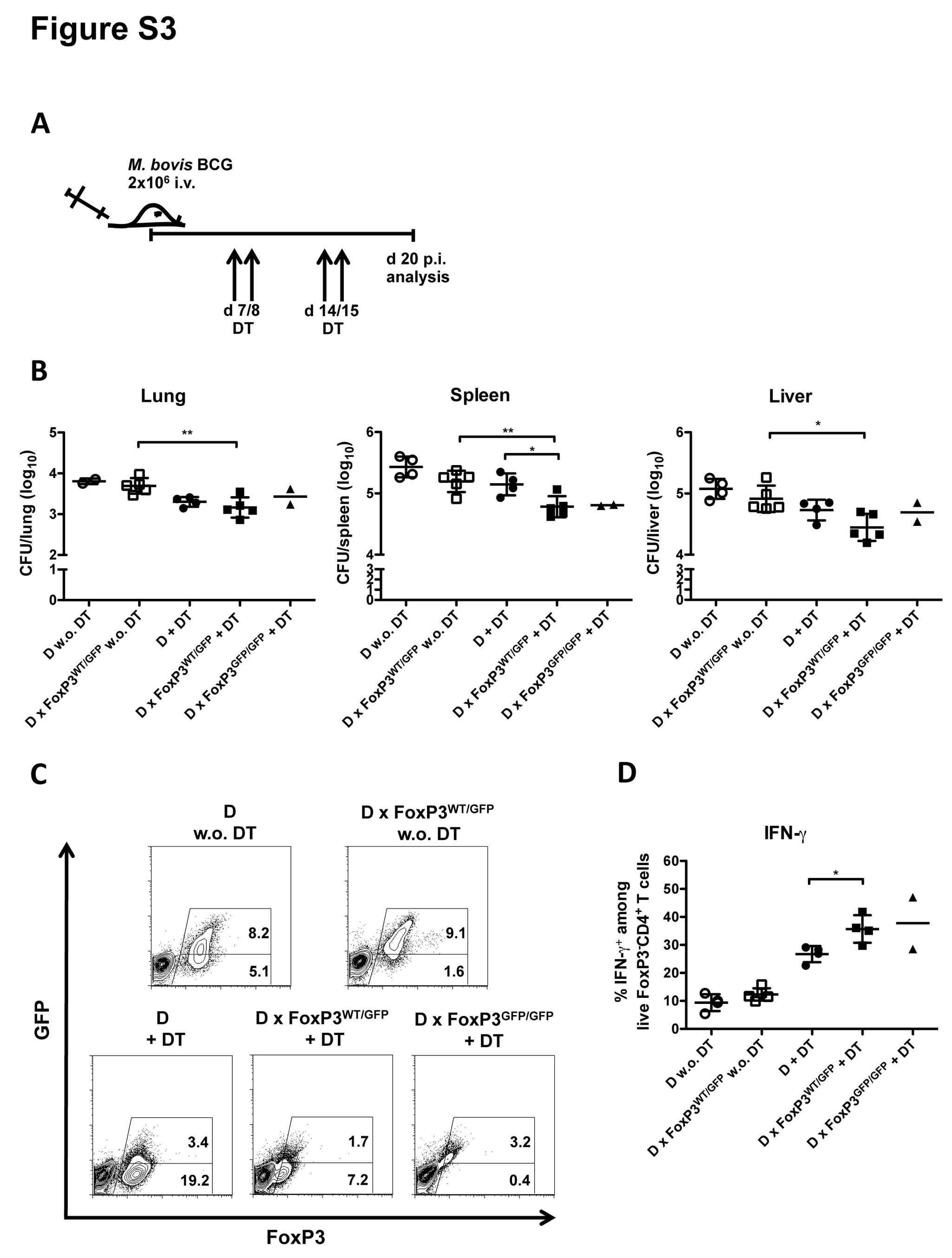

Supplement: Figure S3 — Impaired rebound of Tregs in D× FoxP3GFP mice results in reduced bacterial burden, but is associated with autoimmunity. DEREG mice were infected i.v. with 2×10^6^ CFU M. bovis BCG. Tregs were depleted by DT administration on days 7/8 and 14/15 (black symbols) or not (white symbols). (A) Experimental schema. (B) CFU were assessed in lungs, spleen and liver on day 20 p.i.. (C) Representative FACS-plots of FoxP3- and GFP-expression by CD4+ T cells from DEREG, D× FoxP3WT/GFP and D× FoxP3GFP/GFP mice on day 20 p.i. treated with two rounds of DT or non-treated. (D) Frequencies of intracellular IFN-γ production by live FoxP3−CD4+ T cells in the spleen of DT-treated or untreated DEREG (D, dots), D× FoxP3WT/GFP (squares) and D× FoxP3GFP/GFP (triangles) mice. (B,D) Each symbol shows an individual mouse. Data represent mean ± SD of 2–5 mice per group. N = 1. Statistical analysis: Mann-Whitney-U-Test. *p<0.05; **p<0.01; and ***p<0.001. (TIF) [file pone.0102804.s003.tif]
